# The Pathophysiological Interrelationship Between Metabolic Dysfunction-Associated Steatotic Liver Disease and Cardiovascular Disease

**DOI:** 10.3390/antiox15060710

**Published:** 2026-06-03

**Authors:** Adrián Róbert Gál, István Szokodi, Zoltán Vizvári, Nina Győrfi, András Vereczkei, Zoltán Petykó, Zoltán Karádi, Attila Tóth

**Affiliations:** 1 Institute of Physiology, Medical School, University of Pécs, 7624 Pécs, Hungary; istvan.szokodi@pte.hu (I.S.); gyorfi.nina@pte.hu (N.G.); zoltan.petyko@aok.pte.hu (Z.P.); zoltan.karadi@aok.pte.hu (Z.K.); 2 Multidisciplinary Medical and Engineering Cellular Bioimpedance Research Group, János Szentágothai Research Center, University of Pécs, 7624 Pécs, Hungary; vizvariz@gmail.com; 3 Heart Institute, Medical School, University of Pécs, 7624 Pécs, Hungary; 4 Molecular Cardiology Research Group, János Szentágothai Research Center, University of Pécs, 7624 Pécs, Hungary; 5 John von Neumann Faculty of Informatics, Óbuda University, 1044 Budapest, Hungary; 6 Bioimpedance Technologies Research Center, University Research and Innovation Center, Óbuda University, 1034 Budapest, Hungary; 7 Department of Surgery, Clinical Center, Medical School, University of Pécs, 7624 Pécs, Hungary; vereczkei.andras@pte.hu

**Keywords:** MASLD, cardiovascular disease, liver-heart axis, oxidative stress, glutathione, inflammation

## Abstract

Metabolic dysfunction–associated steatotic liver disease (MASLD) is a highly prevalent multisystem disorder and is strongly associated with increased cardiovascular risk. Cardiovascular diseases represent the leading cause of mortality in this population. As the hepatic manifestation of systemic metabolic dysfunction, MASLD is initiated by excess lipid accumulation driven by increased dietary fatty acid intake and accelerated de novo lipogenesis. This triglyceride overload induces lipotoxicity, triggering hepatocellular injury, immune activation, and mitochondrial dysfunction. Excessive mitochondrial reactive oxygen species (ROS) generation acts as a critical second hit, promoting inflammatory cytokine production and disease progression. Beyond lipid dysregulation, impaired hepatic insulin signaling leads to hyperglycemia and compensatory hyperinsulinemia, further stimulating lipogenesis and reinforcing a self-perpetuating metabolic cycle. Persistent ROS production overwhelms antioxidant defenses and depletes hepatic glutathione (GSH), resulting in systemic redox imbalance. These disturbances extend beyond the liver, contributing to atherogenic dyslipidemia and chronic inflammation. In parallel, gut dysbiosis and increased intestinal permeability amplify immune activation. Reduced circulating GSH further weakens systemic antioxidant capacity; oxidative stress may represent a central mechanistic link between MASLD and CVD. In concert with metabolic and inflammatory mediators, ROS disrupt pathways governing vascular and myocardial homeostasis, leading to coronary atherosclerosis, microvascular dysfunction, left ventricular remodeling, hypertrophy, and impaired relaxation. Clinically, this translates into an increased burden of coronary artery disease and heart failure, particularly heart failure with preserved ejection fraction. Given this integrated pathophysiology, early identification of subclinical cardiovascular involvement is essential. We highlight emerging biomarkers, advocate for multidisciplinary screening strategies, and discuss integrated pharmacological approaches targeting shared metabolic pathways. Recognizing MASLD as a cardiovascular risk amplifier is critical for improving risk stratification and enabling the development of effective, co-targeted therapeutic strategies.

## 1. Introduction

Metabolic dysfunction-associated steatotic liver disease (MASLD) is defined by the pathological accumulation of triglycerides within hepatocytes, typically exceeding 5% of liver volume as assessed by imaging modalities such as magnetic resonance imaging (MRI). Hepatic lipid content derives predominantly from circulating albumin-bound fatty acids (~60%), with additional contributions from dietary sources (~15%) and de novo lipogenesis (~25%) [1,2,3]. Clinically, MASLD is diagnosed by the presence of liver steatosis in the absence of secondary causes, including significant alcohol consumption (≥30 g/day in men, ≥20 g/day in women), viral infections (hepatitis C virus), or inherited metabolic disorders such as Wilson’s disease or α1-antitrypsin deficiency [4].

MASLD is closely linked to metabolic syndrome and is widely regarded as its hepatic manifestation. It frequently coexists with obesity, type 2 diabetes mellitus, and dyslipidemia, reflecting a shared pathophysiological substrate of insulin resistance and metabolic dysregulation [5,6]. Disease progression in MASLD is influenced by a combination of demographic, genetic, and environmental factors, including aging, genetic susceptibility, chronic low-grade inflammation, gut dysbiosis, and metabolic abnormalities such as insulin resistance and hyperinsulinemia [7,8,9,10].

The relationship between MASLD and cardiovascular disease (CVD) is complex and multifactorial, underpinned by shared mechanisms including insulin resistance, atherogenic dyslipidemia, endothelial dysfunction, and chronic low-grade systemic inflammation [11,12]. Beyond its well-established vascular effects, MASLD also directly impacts cardiac structure and function, with accumulating evidence demonstrating associations with left ventricular remodeling, increased myocardial stiffness, and impaired diastolic relaxation—even in the absence of overt cardiovascular disease—ultimately predisposing patients to heart failure, particularly heart failure with preserved ejection fraction (HFpEF) [13,14]. These alterations are mediated by a combination of lipotoxicity, myocardial energy dysregulation, microvascular dysfunction, and systemic inflammatory signaling, and are further amplified by liver-derived bioactive mediators, including inflammatory cytokines and hepatokines, which modulate vascular function, myocardial remodeling, metabolic regulation, and redox homeostasis [11,12,13,14]. Through these interconnected pathways, MASLD exerts integrated effects on both vascular integrity and cardiac performance, reinforcing the concept of a bidirectional liver–heart axis.

Despite its high prevalence and clinical relevance, MASLD often remains undiagnosed in routine practice due to its largely asymptomatic nature and the lack of systematic screening in cardiology and internal medicine settings [11,15]. This under-recognition represents a critical gap, given that MASLD is increasingly acknowledged not merely as a hepatic condition but as a clinically meaningful cardiovascular risk enhancer. Among hepatic features, liver fibrosis has emerged as the strongest predictor of adverse cardiovascular outcomes [16,17].

In this review, we explore the molecular interplay between the liver and the cardiovascular system, emphasizing the concept of the liver–heart axis. We argue that MASLD contributes independently to CVD through shared metabolic, inflammatory, and endocrine pathways that affect both vascular and myocardial compartments. Failure to incorporate liver disease severity into cardiovascular risk assessment may, therefore, represent a missed opportunity for early identification and intervention in high-risk individuals.

To provide a comprehensive and up-to-date synthesis, this narrative review is based on a targeted search of PubMed and Google Scholar databases using combinations of the following keywords: “metabolic dysfunction-associated steatotic liver disease”, “non-alcoholic fatty liver disease”, “MASLD”, “NAFLD”, “cardiovascular disease”, “atherosclerosis”, and “heart failure”. Priority was given to large population-based cohort studies, randomized controlled studies, meta-analyses, and major guidelines, and we emphasized several preclinical studies and consensus statements published between 2000 and 2026. The aforementioned databases were last screened on 11 May 2026, with a particular emphasis on recent studies that reflect contemporary definitions of MASLD. Reference lists of relevant articles were also screened to identify additional key publications.

## 2. Molecular Drivers of Chronic Low-Grade Inflammation

In 2023, the Nomenclature Consensus Group redefined non-alcoholic fatty liver disease (NAFLD) as MASLD, reflecting the central role of systemic metabolic dysfunction in its pathogenesis [18]. The “multiple parallel hits” model conceptualizes MASLD as the consequence of converging insults, including insulin resistance, lipotoxicity, oxidative and endoplasmic reticulum stress, and gut dysbiosis [1,19]. Collectively, these interconnected mechanisms drive hepatic steatosis and establish a systemic pro-inflammatory and pro-atherogenic milieu, thereby promoting the development and progression of cardiovascular disease (CVD) [20,21].

**Figure 1:**
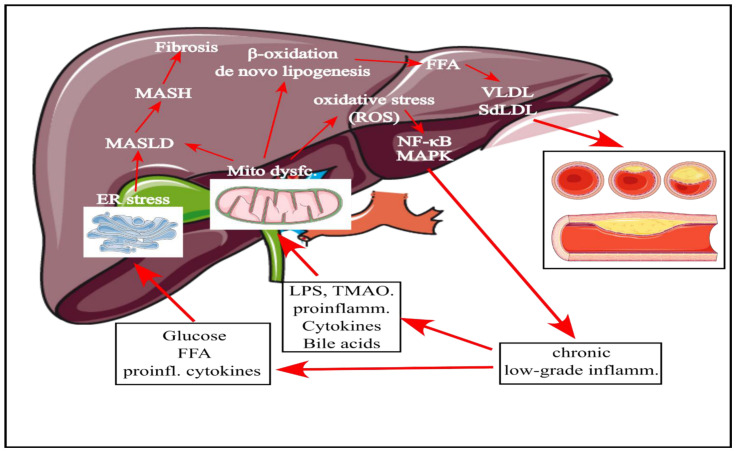
Lipotoxic–oxidative axis in MASLD. Abbreviations: LPS: lipopolysaccharide; ER: endoplasmic reticulum; Mito dysfc.: mitochondrial dysfunction; NF-κB: Nuclear Factor Kappa-Light-Chain-Enhancer of Activated B Cells; MAPK: mitogen-activated protein kinase; TMAO: trimethylamine N-oxide.

Central to this metabolic derangement is the expansion of visceral adipose tissue, which increases free fatty acid (FFA) flux to the liver via the portal circulation, thereby promoting hepatic insulin resistance and stimulating de novo lipogenesis. This dysregulated hepatic lipid metabolism accelerates MASLD progression and drives atherogenic dyslipidemia, characterized by elevated triglycerides, increased remnant lipoprotein cholesterol, and a predominance of small dense low-density lipoprotein (sdLDL) particles. These sdLDL particles readily infiltrate the arterial intima, promoting lipid retention and vascular inflammation, thereby facilitating atherosclerotic plaque formation [22] (Figure 1).

Chronic low-grade inflammation represents the cornerstone of both advanced MASLD, including metabolic dysfunction-associated steatohepatitis (MASH), fibrosis, and cirrhosis (Figure 1), and extrahepatic manifestations, such as type 2 diabetes mellitus, chronic kidney disease, and CVD [23,24]. Collectively, these inflammation-mediated conditions account for nearly half of global mortality [25]. In MASLD, persistent subclinical inflammation is characterized by elevated circulating biomarkers, including tumor necrosis factor-α (TNF-α), interleukin-6 (IL-6), C-reactive protein (CRP), and fibrinogen. This inflammation, coupled with oxidative stress, impairs endothelial function and promotes subclinical atherosclerosis [26]. A robust body of evidence now underscores a stringent association between MASLD and a broad spectrum of cardiovascular pathologies. Consistent with these mechanistic insights, MASLD is a potent correlate of subclinical vascular injury, manifesting as increased carotid intima–media thickness, heightened arterial stiffness, accelerated coronary artery calcification, and attenuated flow-mediated dilation [27,28,29]. Emerging longitudinal data further establish MASLD as an independent predictor of major adverse cardiovascular events (MACE), encompassing myocardial infarction, ischemic stroke, coronary revascularization, and cardiovascular death. Consequently, MASLD is increasingly recognized as a key driver of cardiometabolic risk beyond traditional factors [30,31,32].

### 2.1. The Lipotoxic–Oxidative Axis: From Organelle Stress to Systemic Inflammation

#### 2.1.1. Ectopic Fat and the Pathogenesis of Atherogenic Dyslipidemia

A hallmark of cardiometabolic dysfunction is the expansion of visceral adipose tissue and the pathological accumulation of dysfunctional ectopic fat within the liver, pancreas, peri- and epicardium, kidneys, and skeletal muscle. These ectopic deposits, together with their lipotoxic metabolites, initiate a cascade of organelle dysfunction and redox imbalance that drives cellular injury and systemic metabolic perturbation [33,34,35].

The onset of MASLD is rooted in a primary imbalance between lipid acquisition and disposal. This metabolic disequilibrium is characterized by an increased flux of circulating lipids, accelerated hepatic de novo lipogenesis, impaired fatty acid oxidation, and dysregulated very low-density lipoprotein (VLDL) secretion [36,37]. While VLDL overproduction initially serves as a compensatory mechanism to export excess hepatic lipids to peripheral tissues [38], it inadvertently generates atherogenic dyslipidemia, defined by reduced high-density lipoprotein (HDL) levels and increased concentrations of triglyceride-rich lipoproteins, intermediate-density lipoproteins (IDL), and sdLDL particles (Figure 1) [10,38,39].

#### 2.1.2. The TLR–NLRP3 Signaling Axis and Vascular Immune Activation

Apolipoprotein-B-containing lipoproteins are the primary drivers of atherosclerosis [10]. Their subendothelial retention and oxidative modification generate damage-associated molecular patterns (DAMPs) that activate Toll-like receptors (TLRs), particularly TLR2 and TLR4, thereby initiating sterile innate immune responses [10,40,41]. A key mechanistic link in preclinical studies shows that this process is apolipoprotein C3 (ApoC3), as ApoC3-enriched triglyceride-rich lipoproteins promote TLR2/4 dimerization and downstream activation of the NLRP3 inflammasome (NOD-like receptor family, pyrin domain-containing protein 3) [10,40]. This cascade culminates in caspase-1 (interleukin-1β converting enzyme) activation and proteolytic maturation of pro-inflammatory cytokines, including IL-1β, thereby initiating the IL-1β–IL-6–CRP cascade that drives vascular inflammation and atherosclerotic CVD [42]. Collectively, the TLR–NLRP3 signaling axis links the atherogenic lipid milieu of MASLD to vascular immune activation [10].

In parallel, enhanced hepatic de novo lipogenesis alters lipoprotein composition by enriching VLDL particles with saturated fatty acids, particularly palmitate [10,43]. These lipotoxic species directly induce TLR2/4 signaling, further reinforcing inflammation [9,44]. Consequently, elevated circulating levels of palmitic (C 16:0) and palmitoleic (C 16:1 *n*-7) acids are recognized as independent predictors of increased cardiovascular mortality [45,46].

#### 2.1.3. Subcellular Crisis: ER Stress and Mitochondrial Dysfunction

At the cellular level, lipid overload disrupts organelle homeostasis, particularly within the endoplasmic reticulum (ER) and mitochondria. ER stress arises from the accumulation of misfolded proteins and activates the unfolded protein response via PERK–eIF2α–ATF4, IRE1–XBP1, and ATF6 pathways. While initially adaptive, sustained ER stress promotes steatosis and disease progression, with transcriptional regulators such as FOXA3 linking ER stress to lipid accumulation [47]. In parallel, mitochondrial dysfunction impairs fatty acid β-oxidation, leading to intracellular lipid accumulation and reinforcing lipotoxicity (Table 1). Although early MASLD may feature compensatory increases in mitochondrial respiration, this becomes maladaptive, resulting in excessive reactive oxygen species (ROS) generation and mitochondrial injury (Figure 1) [48].

#### 2.1.4. Redox Imbalance and the Glutathione Bridge

Within this framework, oxidative stress emerges as a central amplifier of liver–heart crosstalk. In MASLD, lipid overload and mitochondrial dysfunction drive ROS production, leading to lipid peroxidation, oxidative DNA damage, and inflammatory activation that promotes fibrosis and insulin resistance [12,69,70]. Systemically, oxidative stress contributes to endothelial dysfunction, lipid oxidation, and plaque progression, ultimately increasing the risk of hypertension, myocardial infarction, and heart failure [71,72]. Importantly, oxidative stress both results from and perpetuates lipotoxic injury, creating a self-reinforcing cycle [70] (Figure 1).

In vivo (laboratory mice) performed experiments showed that at the molecular level, ROS amplify inflammatory signaling through TLR4/NF-κB and NLRP3/NF-κB pathways [73,74], while chemokine axes (e.g., CXCR3–CXCL9/10/11 and CXCR6–CXCL16) regulate immune cell recruitment, macrophage activation, and fibrogenesis [49,66,75,76,77]. Concurrent impairment of antioxidant defenses further exacerbates redox imbalance.

**Figure 2:**
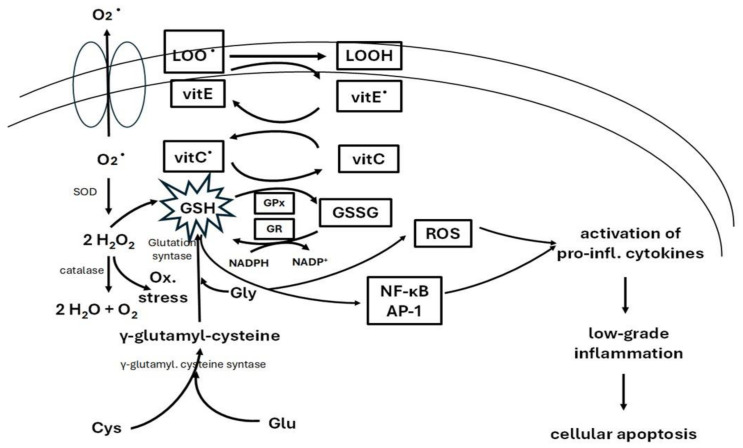
Central role of glutathione in redox homeostasis. Abbreviations: SOD, superoxide dismutase; Cys, cysteine; Glu, glutamine; Gly, glycine; GSSG, glutathione disulfide; NADPH, nicotinamide dinucleotide phosphate (reduced state); NADP^+^, nicotinamide dinucleotide phosphate (oxidized state); GPx, glutathione peroxidase; GR, glutathione reductase; AP-1, activator protein-1; LOO., lipid hydroperoxyl radical; LOOH, lipid hydroperoxide, O_2_^.−^, superoxide anion, H_2_O_2,_ hydrogen peroxide, H_2_O, water, O_2,_ oxygen.

Glutathione (GSH), a key intracellular antioxidant synthesized from glutamate, cysteine, and glycine via glutamate–cysteine ligase and glutathione synthetase, is reduced in MASLD, particularly in the presence of insulin resistance [78,79,80]. As the liver is the principal source of systemic GSH, impaired hepatic synthesis disrupts inter-organ redox homeostasis, contributing to increased cardiovascular risk [81,82]. Circulating GSH is recycled via γ-glutamyl transferase (γ-GT), and elevated serum γ-GT levels serve as a clinical marker of oxidative stress associated with metabolic disorders and CVD (Figure 2) [83]. Consistently, broader antioxidant defenses, including superoxide dismutase and catalase, are diminished in MASLD, while dysregulation of the Keap1–Nrf2 pathway further limits adaptive antioxidant responses; conversely, Nrf2 activation restores redox balance and attenuates hepatic injury [84,85,86,87,88].

#### 2.1.5. The Glucose–Insulin–Lipid Axis and Selective Insulin Resistance

**Figure 3:**
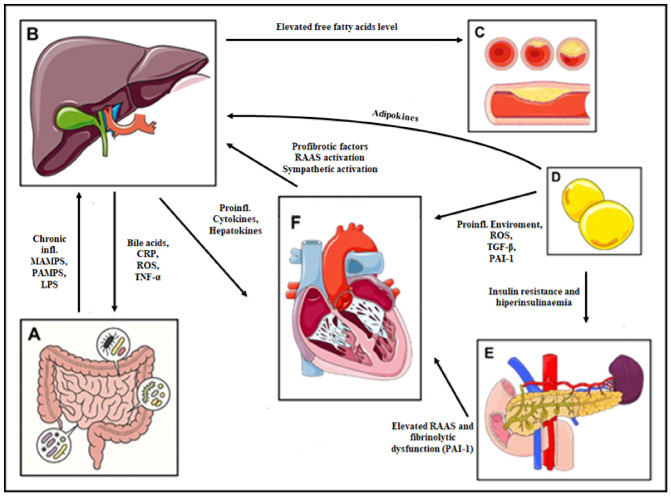
Pathophysiologic mechanisms linking MASLD to CVD. Abbreviation: TGF, transforming growth factor. (**A**): gut dysbiosis, “leaky gut”, (**B**): MASLD, (**C**): atherogen dyslipidemia, (**D**): ectopic adipose tissue, (**E**): β-cell dysfunction + systemic insulin resistance, (**F**): cardiovascular diseases: coronary artery disease, myocardial infarction, heart failure with preserved ejection fraction.

Beyond lipid dysregulation and oxidative imbalance, hepatic insulin resistance and disordered glucose metabolism represent core features of MASLD and key pathophysiological links to CVD [89,90]. Glucose dysregulation is closely associated with chronic low-grade inflammation, visceral adiposity, and ectopic fat accumulation [91]. Notably, lipid deposition within the pancreas plays a critical role in catalyzing β-cell dysfunction and systemic insulin resistance [91,92,93]. This state is typically accompanied by compensatory hyperinsulinemia, which, together with increased hepatic glucose production, sustains hyperglycemia and elevated circulating FFA levels, establishing a self-reinforcing cycle of metabolic dysfunction. In MASLD, this cycle is further amplified by reduced hepatic insulin clearance (Figure 3).

At the molecular level, impaired insulin signaling paradoxically coexists with selective preservation of lipogenic pathways. Activation of sterol regulatory element-binding protein 1c (SREBP-1c) and carbohydrate-responsive element-binding protein (ChREBP) drives the transcription of key lipogenic enzymes, thereby accelerating de novo lipogenesis and reinforcing lipid accumulation. This selective insulin resistance phenotype integrates glucose and lipid dysregulation, linking hepatic steatosis to systemic metabolic impairment [10].

#### 2.1.6. Systemic Consequences and Dietary Drivers

Insulin resistance and impaired insulin signaling are primary drivers of atherogenesis, contributing to both plaque formation and instability. Chronic hyperglycemia promotes oxidative stress and activates multiple inflammatory cascades, including the inflammasome and advanced glycation end product-mediated vascular injury [9,92,93]. In parallel, insulin resistance triggers maladaptive neurohumoral responses, such as the activation of the renin–angiotensin–aldosterone system (RAAS), and impairs fibrinolysis through elevated plasminogen activator inhibitor-1 (PAI-1) levels. These systemic changes are often accompanied by cardiac autonomic neuropathy, which exacerbates endothelial dysfunction and promotes systolic/diastolic impairment, and increases susceptibility to arrhythmias [9,94,95,96,97] (Figure 3).

Dietary factors represent an important extrahepatic driver of MASLD-related metabolic and inflammatory dysregulation. Western-style diets, rich in trans-unsaturated fatty acids, activate the SREBP cleavage-activating protein (SCAP)-SREBP axis, promoting cholesterologenesis and hepatic steatosis [98]. Excessive sugar ingestion, particularly fructose, drives hepatic lipogenesis through activation of ChREBP and can induce steatosis even independent of canonical insulin-mediated pathways [99,100]. In parallel, fructose metabolism promotes the generation of advanced glycation end products and activates nuclear factor-κB (NF-κB)-mediated inflammatory signaling, thereby exacerbating oxidative stress and hepatocellular injury [101]. This signaling pathway represents a promising therapeutic target, as its inhibition has been shown to induce fatty liver disease regression [102]. Collectively, excessive carbohydrate intake, especially fructose, impairs insulin sensitivity, enhances de novo lipogenesis, and disrupts redox homeostasis, further accelerating disease progression [102,103,104].

### 2.2. The Adipokine–Liver Axis: Endocrine Mediators of Cardiometabolic Dysfunction

Insulin resistance is a central driver of hepatic oxidative stress, impairing mitochondrial β-oxidation of free fatty acids and disrupting lipoprotein lipase activity [105]. Although its association with MASLD is well established, the directionality of this relationship, whether insulin resistance precedes or arises as a consequence of hepatic steatosis, remains incompletely resolved. Nonetheless, experimental evidence consistently demonstrates a positive correlation between insulin resistance and hepatic inflammatory markers, alongside a robust inverse association with HDL cholesterol levels [106,107,108,109].

Adipose tissue distribution is sexually dimorphic and tightly regulated by endocrine factors, and this hormonal control plays a pivotal role in MASLD pathogenesis through adipokine secretion. These bioactive mediators regulate metabolic homeostasis, inflammation, and insulin sensitivity, whereas their dysregulation, often driven by altered fat distribution, promotes obesity-related low-grade inflammation and progression of metabolic syndrome (Table 2) [109].

Among adipokines, adiponectin exerts anti-inflammatory and insulin-sensitizing effects by activating 5′AMP-activated protein kinase (AMPK), thereby inhibiting acetyl-CoA carboxylase and fatty acid synthesis, reducing hepatic lipid influx; its circulating levels are inversely associated with insulin resistance (Table 2) [110]. In contrast, leptin, a key regulator of energy balance and satiety, becomes dysregulated in obesity, where leptin resistance and hyperleptinemia contribute to steatosis, insulin resistance, and pro-inflammatory and pro-fibrotic signaling (Table 2) [111]. Resistin promotes inflammation and metabolic dysfunction via activation of the NF-κB pathway and modulation of mitochondrial activity, linking innate immunity to insulin resistance (Table 2) [112]. Visfatin further exacerbates inflammation and insulin resistance through activation of TLR4 and downstream JAK2/STAT3 and NF-κB signaling pathways (Table 2) [113,114].

Additional adipokines contribute to the complex metabolic milieu: adipsin (complement factor D) is involved in lipid storage and glucose metabolism and has been associated with insulin resistance and systemic inflammation, with potential utility as a biomarker in MASLD [115,116]; omentin (intelectin-1) enhances insulin signaling via the PI3K–Akt pathway and is inversely related to insulin resistance, although its role in hepatic injury remains context-dependent [117,118]; and vaspin, a serine protease inhibitor, may exert compensatory insulin-sensitizing effects in states of metabolic stress, correlating with both inflammation and insulin resistance (Table 2) [119].

Collectively, MASLD is characterized by adipose tissue dysfunction and a profoundly altered adipokine profile, with reduced adiponectin and elevated leptin and resistin levels. This imbalance promotes hepatic lipid accumulation, systemic inflammation, and vascular dysfunction, thereby contributing to a pro-atherogenic environment and increased cardiovascular risk [120,121,122].

**Table 2:** Circulating adipokines in MASLD and CVD: Evidence from clinical cohorts.

Adipokine	Type	Primary Biological Function	Impact on MASLD	Impact on CVD
Adiponectin	Peptide hormone	• ↑ Insulin sensitivity • ↑ Fatty acid oxidation • Anti-inflammatory effects	• ↓ Serum adiponectin: associated with ↑ risk of MASLD [116] • ↓ Circulating adiponectin: correlates with ↑ risk of MASLD development [123] • Serum adiponectin: ↓↓ in MASH vs. ↓ in MASLD [124]	• ↓ Plasma adiponectin: associated with ↑ CAD risk [125] • ↓ Plasma adiponectin: associated with ↑ risk of myocardial infarction in men without prior CVD [126]
				
Leptin	Peptide hormone	• Regulation of long-term energy balance, appetite, and satiety	• ↑ Serum leptin in MASLD [124] • ↑ Serum leptin in high-altitude MASLD patients (independent of obesity) [123]	• ↑ Serum leptin in advanced chronic heart failure [127] • ↑ Plasma leptin: associated with ↑ risk of heart failure in men without pre-existing CAD [128] • ↑ Serum leptin in the general population: predicts ↑ risk of incident heart failure [129]
				
Resistin	Cysteine-rich peptide hormone	• Promotes insulin resistance • ↑ Pro-inflammatory cytokine synthesis • Modulates macrophage activation	• Serum resistin: ↑ in MASLD vs. controls; ↓ in MASH vs. controls [130]	• ↑ Serum resistin: independently predicts ↑ risk of myocardial infarction in the general population [131] • ↑ Serum resistin in the general population: predicts ↑ risk of cardiac death, and incident heart failure [129] • Serum resistin: ↑ in heart failure vs. controls; correlates with disease severity and ↑ risk of adverse events (cardiac death or re-hospitalization for worsening heart failure) [132]
				
Visfatin	Dual-function pleiotropic protein (extracellular enzyme/cytokine)	• ↑ Glucose uptake in adipocytes • ↓ Hepatic glucose release • Pre-adipocytes: ↑ triglyceride synthesis and intracellular accumulation • Regulates rate-limiting intracellular NAD+ biosynthesis	• ↓ Serum visfatin: associated with ↑ risk of MASLD [116] • Serum visfatin: similar in MASLD vs. controls [133]	• Serum visfatin: ↑ in myocardial infarction vs. controls; associated with ↑ incidence of adverse events (cardiac death, nonfatal recurrent myocardial infarction, or hospitalization for heart failure) [134]
				
Adipsin	Enzyme (serine protease)	• Rate-limiting catalyst of the alternative complement pathway • Generates C3a → activates pancreatic β-cell C3aR to ↑ glucose-dependent insulin secretion [135]	• ↑ Serum adipsin: associated with ↑ risk of MASLD [116] • Serum adipsin: similar in MASLD vs. controls [136] • ↑ Serum adipsin in MASLD [137]	• ↑ Serum adipsin in CAD: ↑ risk of all-cause mortality, rehospitalization, and fatal myocardial infarction [138] • ↑ Serum adipsin in the general population: predicts ↑ risk of all-cause mortality, cardiac death, and incident heart failure [129]
				
Omentin	Carbohydrate-binding protein	• ↑ Insulin-mediated glucose uptake • Microbial clearance	• ↑ Serum omentin in MASLD • ↑ Serum omentin: independently predicts hepatocyte ballooning [118]	• ↑ Serum omentin-1 vs. heart failure risk: modified by pre-existing CAD: - without CAD: linear association - with CAD: U-shaped association [139] • ↑ Serum omentin-1: predicts ↑ risk of cardiovascular events [140]
				
Vaspin	Glycoprotein class A member of serine protease inhibitor (serpin) family	• ↑ Insulin sensitivity • ↑ Anti-inflammatory activity	• Serum vaspin: similar in MASLD vs. controls [141]	• ↓ Serum vaspin: independently predicts ↑ risk of adverse cardiac events following myocardial infarction [142]

Abbreviations: CAD: coronary artery disease, CVD: cardiovascular disease, MASLD: metabolic dysfunction-associated steatosis liver disease, MASH: metabolic dysfunction-associated steatohepatitis, ↑: increase, ↓: decrease.

#### Key Hepatokines and Their Physiological/Pathophysiological Roles

Emerging evidence highlights the role of hepatokines in linking MASLD to CVD. Hepatokines are liver-derived, hormone-like proteins that regulate systemic metabolism and vascular function, thereby contributing to cardiometabolic risk [143,144].

➢ Fibroblast growth factor 21 (FGF-21): A key metabolic regulator involved in glycemic control, energy expenditure, and body weight homeostasis. Experimental data suggest that FGF-21 can attenuate hepatic steatosis and exert cardioprotective effects via blood pressure reduction and mitigation of oxidative stress. Notably, FGF-21 is also produced by cardiomyocytes in response to oxidative stress, supporting bidirectional heart–liver cross-talk. In MASLD, circulating FGF-21 levels are elevated, reflecting hepatic stress and correlating with insulin resistance and subclinical atherosclerosis [145,146].
➢ Fetuin-A (α2HS-glycoprotein): A hepatokine that inhibits transforming growth factor (TGF)-β1 signaling. In MASLD, increased fetuin-A levels contribute to insulin resistance by impairing insulin receptor signaling. In addition, fetuin-A promotes vascular calcification, thereby linking MASLD to CVD progression [145,147].
➢ Selenoprotein P and Angiopoietin-like protein 8 (ANGPTL8): Secreted primarily by hepatocytes, these proteins are central to the regulation of lipid metabolism. Their elevation in MASLD is closely linked to endothelial dysfunction and impaired lipid clearance, further predisposing patients to atherogenic progression [148].

### 2.3. Gut Dysbiosis

Disruption of gut microbial homeostasis, referred to as dysbiosis, represents an important contributor to cardiometabolic dysfunction by perturbing both metabolic and immune pathways [149]. A defining feature of dysbiosis is impairment of the intestinal barrier, resulting in increased mucosal permeability (“leaky gut”). This facilitates the translocation of microbial components and metabolites, including microbe- or pathogen-associated molecular patterns such as lipopolysaccharide (LPS) and peptidoglycans, as well as DAMPs released from injured enterocytes, into the systemic circulation. These signals activate innate immune pathways and promote a chronic, low-grade inflammatory state that represents a key mechanistic link between gut dysbiosis and cardiometabolic diseases, including atherosclerosis [9,36,150,151] (Figure 3).

Within the liver, portal influx of microbial products alters the balance between pro- and anti-inflammatory signaling, promoting hepatic inflammation, lipotoxicity, and progression toward MASH [152]. Beyond immune activation, gut microbiota-derived metabolites exert pleiotropic effects on host metabolism and cardiovascular risk. Short-chain fatty acids, primary and secondary bile acids, or trimethylamine N-oxide (TMAO) are among the most extensively studied mediators in this context [36,150].

Trimethylamine, generated by microbial metabolism of dietary choline or L-carnitine, is subsequently oxidized in the liver to TMAO [27]. TMAO exerts multiple pro-atherogenic and pro-thrombotic effects, including promotion of macrophage foam cell formation, impairment of reverse cholesterol transport, and enhancement of endothelial dysfunction and platelet activation [153]. These effects are mediated, at least in part, by increased expression of pro-inflammatory cytokines (e.g., IL-6, CRP, TNF-α) and augmented ROS production, alongside reduced nitric oxide bioavailability [154]. Consistent with this, bacterial DNA has been detected within human atherosclerotic plaques [155], and LPS derived from Gram-negative bacteria can directly interact with the endothelium to trigger inflammatory signaling and vascular dysfunction [153,156].

Conversely, certain microbiota-derived metabolites exert protective effects. Short-chain fatty acids, produced through bacterial fermentation of complex carbohydrates, improve lipid metabolism by decreasing LDL and triglyceride levels, while increasing HDL, thereby attenuating inflammation and improving insulin sensitivity [157]. These effects are mediated in part through activation of G protein-coupled receptors (GPR41 and GPR43), which stimulate the release of glucagon-like peptide-1 (GLP-1) and peptide YY, contributing to the regulation of glucose homeostasis and appetite [158].

## 3. Cardiovascular Pathophysiological Manifestations and the Liver–Heart Axis in MASLD

### 3.1. MASLD as a Systemic Multisystem Disease and Cardiovascular Risk Amplifier

MASLD is increasingly recognized as a systemic, progressive disorder that extends beyond the liver and acts as an independent risk factor for CVD, irrespective of traditional metabolic comorbidities [159,160]. While liver-related complications remain clinically relevant, CVD represents the leading cause of morbidity and mortality in this population. Patients with MASLD exhibit an increased risk of acute cardiovascular events, including acute coronary syndromes, stroke, and malignant arrhythmias, across all disease stages [13,14,87].

Accumulating evidence indicates that MASLD promotes accelerated coronary atherosclerosis and adversely affects myocardial structure and function, contributing to left ventricular remodeling, hypertrophy, and impaired relaxation [161]. These alterations predispose to heart failure, particularly heart failure with preserved ejection fraction (HFpEF), which has emerged as the dominant phenotype in contemporary populations and represents a major global healthcare burden [13,162,163,164,165,166].

Recognizing MASLD as a multisystem disease underscores the need for integrated clinical management targeting both hepatic and cardiovascular risk factors. Early identification of subclinical cardiovascular involvement is critical, as the coexistence of these pathologies accelerates disease progression and worsens clinical outcomes. A deeper understanding of the shared and intertwined pathophysiological mechanisms offers a foundation for improved risk stratification and the development of targeted therapeutic strategies [167,168].

### 3.2. Endothelial Dysfunction and Prothrombotic Imbalance

Endothelial dysfunction represents an early and pivotal event in atherogenesis and is a defining feature of cardiometabolic disease [169]. In MASLD, it arises from the convergence of oxidative stress, lipoprotein-driven vascular inflammation, and selective vascular insulin resistance—mechanisms detailed in preceding sections. A key hallmark is reduced bioavailability of nitric oxide, a critical mediator of vascular homeostasis, leading to impaired vasodilation, increased vascular tone, and enhanced platelet activation [9,36,40,90,170].

Circulating biomarkers further reflect this dysfunctional state. Asymmetric dimethyl arginine (ADMA), an endogenous antagonist of nitric oxide synthase, is elevated in MASLD due to impaired hepatic metabolism, resulting in diminished nitric oxide production and altered vasomotor regulation [36,40,90]. Similarly, hyperhomocysteinemia contributes to endothelial injury by promoting oxidative stress, partly through depletion of glutathione stores, thereby linking redox imbalance to vascular dysfunction [40,90].

Endothelial injury is accompanied by a procoagulant shift that further accelerates atherothrombosis.

MASLD is associated with increased levels of coagulation factors (e.g., FVIII, FIX, FXI, FXII), fibrinogen, von Willebrand factor, and PAI-1, alongside reduced anticoagulant capacity, including lower anti-thrombin III levels [40,90,171]. Elevated levels of vascular endothelial growth factor reflect active angiogenesis and vascular remodeling, processes closely linked to plaque progression and instability [172].

### 3.3. Epicardial and Pericardial Adipose Tissue: A Local Mediator of Cardiac Injury

Beyond hepatic pathology, ectopic adipose tissue accumulation within and around the heart represents a critical link between MASLD and CVD [173]. Cardiac adipose tissue is anatomically divided into intrapericardial (epicardial) and extrapericardial compartments, separated by the pericardium. Epicardial adipose tissue, located between the myocardium and the visceral pericardium, is of particular pathophysiological relevance due to its direct anatomical and microcirculatory continuity with the myocardium [159,174].

Under physiological conditions, epicardial adipose tissue exerts cardioprotective effects by supplying fatty acids and secreting anti-inflammatory mediators such as adiponectin [159,174]. However, in MASLD and related metabolic disorders, epicardial adipose tissue undergoes a phenotypic shift toward a pro-inflammatory state, characterized by increased secretion of pro-inflammatory cytokines including TNF-α, IL-1, IL-6, leptin, and resistin. This promotes macrophage infiltration, microvascular inflammation, and activation of pro-fibrotic mechanisms within the myocardium [159,175].

These paracrine interactions induce structural and functional cardiac alterations, including myocardial fibrosis, impaired coronary artery integrity, and arrhythmogenic remodeling. Clinically, this contributes to coronary artery disease, atrial fibrillation, and heart failure, particularly HFpEF [159,175]. In addition, extra-pericardial adipose tissue may influence cardiac autonomic regulation and has been associated with disease severity in both MASLD and cardiovascular conditions. The release of pro-inflammatory mediators into the systemic circulation further amplifies the global cardiometabolic inflammatory burden, reinforcing a vicious cycle of ectopic fat accumulation and organ dysfunction [160,176,177,178].

### 3.4. Mechanistic Pathways Linking MASLD to Heart Failure

The bidirectional relationship between MASLD and CVD is mediated by interconnected mechanisms that collectively drive cardiac dysfunction.

#### 3.4.1. Metabolic Dysfunction and Insulin Resistance

Insulin resistance remains a central driver, promoting increased flux of FFAs from adipose tissue and enhanced hepatic de novo lipogenesis, leading to systemic lipotoxicity [14]. The liver contributes to this pathogenic milieu through the release of pro-inflammatory and pro-thrombotic mediators, including fibrinogen, TGF-β, PAI-1, IL-6, TNF-α, and ROS, which amplify systemic metabolic stress [147]. In the myocardium, excess FFA uptake induces lipotoxic injury, disrupts energy metabolism, and promotes diastolic dysfunction [179,180]. At the cellular level, impaired insulin signaling alters substrate utilization, reduces ATP efficiency, and promotes oxidative stress, mitochondrial dysfunction, and cardiomyocyte apoptosis [181,182,183]. Concurrent disruption of PI3K/Akt signaling reduces nitric oxide bioavailability and contributes to adverse myocardial remodeling [184].

#### 3.4.2. Chronic Inflammation and Adipose Tissue Dysfunction

Persistent low-grade inflammation, driven by adipose tissue expansion and hepatocellular injury, reinforces oxidative stress and endothelial dysfunction [185,186,187,188,189]. The secretion of inflammatory mediators promotes a pro-atherogenic and pro-thrombotic environment, while epicardial adipose tissue exerts local deleterious effects on myocardial structure and function. These processes collectively increase the risk of coronary artery disease and contribute to myocardial remodeling and HFpEF development [185,190,191,192,193].

#### 3.4.3. Hemodynamic Alterations and Hepatic–Cardiac Cross-Talk

Progressive liver fibrosis alters hepatic vascular architecture, increasing sinusoidal resistance and leading to portal hypertension [185,194]. The subsequent development of portosystemic shunts allows vasoactive molecules to bypass hepatic first-pass clearance, contributing to pulmonary vascular remodeling and systemic hemodynamic dysregulation [195]. These alterations impair preload reserve and reduce exercise capacity [185,196]. In concert with diastolic dysfunction, they lead to reduced peak oxygen consumption. Collectively, these alterations underscore a direct pathophysiological link between hepatic dysfunction and cardiovascular impairment [197].

#### 3.4.4. Arterial Dysfunction and Increased Afterload

Hepatic dysfunction, driven by triglyceride deposition and increased parenchymal stiffness, contributes substantially to cardiovascular impairment by elevating systemic vascular resistance [198]. Expansion of perihepatic adipose tissue further promotes endothelial dysfunction, while heightened sympathetic activation augments arterial stiffness and vascular resistance [185]. Epidemiological data provide robust evidence linking MASLD with the pathogenesis of systemic hypertension [199,200]. Increased afterload drives concentric left ventricular hypertrophy, reduces ventricular compliance, and impairs diastolic relaxation [185]. A hallmark of HFpEF in MASLD patients is impaired arterial–ventricular coupling, which limits the cardiovascular reserve under increased metabolic demand and contributes to exercise intolerance. In parallel, MASLD-related microvascular dysfunction further exacerbates myocardial perfusion abnormalities, promoting subclinical myocardial ischemia and progressive cardiac dysfunction [185,201,202].

## 4. Screening and Diagnostic Strategies: A Multidisciplinary Approach to MASLD and CVD Risk

To mitigate underdiagnosis of MASLD and to improve identification of associated cardiovascular risk, a multidisciplinary clinical approach involving cardiologists, hepatologists, endocrinologists, angiologists, and nephrologists is recommended. This approach should focus on the following: (1.) accurate diagnosis and screening for MASLD using non-invasive methods; (2.) systematic CVD screening in patients with confirmed MASLD; (3.) integrated management strategies that combine MASLD treatment with cardiovascular disease prevention [203]. 

The diagnosis of MASLD requires the following:

(a.) Identifying the presence of liver steatosis using radiology techniques or performing a liver biopsy.
(b.) Presence of ≥1 of 5 of the following cardiometabolic criteria:
  1. Body mass index ≥ 25 kg/m^2^ (23 kg/m^2^ in Asians) or waist circumference ≥ 94 cm (for males) or ≥80 cm (for females);
  2. Fasting glucose ≥ 100 mg/dL (5.6 mmol/L) or 2-h post-load glucose level ≥ 140 mg/dL (7.8 mmol/L) or Hba1c ≥ 5.7% (39 mmol/L) or type 2 diabetes or glucose-lowering treatment;
  3. Blood pressure (BP) ≥ 130/85 mm Hg or BP-lowering treatment;
  4. Plasma triglyceride ≥ 150 mg/dL (1.7 mmol/L) or lipid-lowering treatment;
  5. Plasma high-density lipoprotein cholesterol < 40 mg/dL [1.0 mmol/L] (for males) and <50 mg/dL [1.3 mmol/L] (for females) or lipid [204].

Patients diagnosed with MASLD should be systematically evaluated for alternative or coexisting causes of hepatic steatosis, including the exclusion of significant alcohol consumption and other secondary diseases (e.g., viral hepatitis). A comprehensive clinical history should be obtained, including current medications, illicit drug use, and/or detailed alcohol consumption habits. Additional metabolic assessments should include fasting insulin measurement and an oral glucose tolerance test to evaluate the presence of insulin resistance [203].

Screening for MASLD should take into consideration the frequent coexistence of subclinical atherosclerotic CVD. First-line evaluation typically includes biochemical assessment of liver function, such as serum transaminases (ASAT, ALAT) and γ-glutamyl transferase (γ-GT), together with abdominal ultrasonography. Liver ultrasound provides a semi-quantitative assessment of hepatic steatosis; however, the absence of sonographic steatosis does not rule out MASLD, metabolic dysfunction-associated steatohepatitis (MASH), or hepatic fibrosis [203,204]. Several non-imaging-based steatosis scoring systems, including the Fatty Liver Index and Hepatic Steatosis Index, have been proposed to estimate hepatic fat content, although their sensitivity remains limited [205,206]. According to current American Association for the Study of Liver Diseases (AASLD) guidelines, the routine use of liver ultrasonography for steatosis detection is of limited diagnostic value in patients with obesity and/or type 2 diabetes mellitus, in whom hepatic steatosis is highly prevalent. Instead, ultrasonography may be more informative when used to assess liver fibrosis in individuals at increased risk, such as those with type 2 diabetes mellitus, obesity with metabolic complications, or ≥ 2 cardiometabolic risk factors, or when combined with non-invasive fibrosis scores, including the Fibrosis-4 Index (FIB-4) [203,207,208].

MASLD disease activity, encompassing hepatocellular injury (ballooning), inflammation, and fibrosis, can be assessed by the extent of hepatic scarring, which indirectly estimates the risk of progression to cirrhosis. Disease activity has traditionally been evaluated using the NAFLD Activity Score (NAS), a biopsy-based semi-quantitative system that grades hepatic lesions. Although NAS correlates well with metabolic indices such as the homeostasis model assessment of insulin resistance (HOMA-IR) and serum transaminase levels, its prognostic value is limited. In contrast, the fibrosis stage represents the strongest predictor of both liver-related and cardiovascular outcomes [4,203]. For fibrosis risk stratification, non-invasive tests (NITs) such as the NAFLD Fibrosis Score, FIB-4, and aspartate transaminase-to-platelet ratio index (APRI) are recommended as first-line screening tools. These indices enable early risk assessment in MASLD and may reduce the need for liver biopsy. NITs can be classified into serum-based and imaging-based biomarkers, both of which are widely used for diagnosis, prognostication, and monitoring disease progression and treatment response [203]. The FIB-4 score, which incorporates age, ALAT, ASAT, and platelet count, is particularly useful in routine clinical practice. An FIB-4 score < 1.3 has a high negative predictive value for advanced fibrosis and can be used to reliably exclude advanced fibrosis, including in patients with coexisting cardiovascular diseases. Conversely, an FIB-4 score > 2.67 suggests advanced fibrosis and warrants referral to a hepatologist for further evaluation and secondary risk assessment [209].

Second-line NITs include the Enhanced Liver Fibrosis (ELF) test, which is commonly used for prognostication in patients with suspected advanced fibrosis and demonstrates diagnostic performance comparable to vibration-controlled transient elastography (VCTE). An ELF score > 11.3 is a strong predictor of liver-related illnesses and cardiovascular outcomes. Sequential use of primary NIT (FIB-4) followed by a secondary NIT (ELF or VCTE) improves identification of patients at high risk for advanced fibrosis and supports timely referral to specialist care. In cases with a high likelihood of cirrhosis, combined application of serum-based and imaging-based techniques, such as emerging/investigational methods, including VCTE and magnetic resonance elastography (MRE), is recommended [203,204]. MRE provides a highly accurate assessment of liver fibrosis and is typically performed following hepatology consultation. Among first-line imaging methods, in addition to conventional abdominal ultrasonography, acoustic radiation force impulse elastography may be considered, especially in cases with abnormal liver-related serology findings or altered biochemical indices [210].

Screening protocol: More than half of patients with MASLD or MASH are asymptomatic, and liver disease is frequently detected incidentally during routine liver function testing [211]. Screening is, therefore, strongly recommended in individuals with obesity, metabolic syndrome, type 2 diabetes mellitus, or who are at high risk for CVD [212]. FIB-4 is recommended as the first-line point-of-care test for liver fibrosis risk stratification.

- Low cardiometabolic risk + FIB-4 < 1.3: advanced fibrosis can be reliably excluded.
- Intermediate risk + FIB-4 ≥ 1.3: further VCTE assessment is indicated.
- High-risk patients, including those with prediabetes or type 2 diabetes mellitus + high cardiometabolic risk: sequential evaluation should be performed using secondary NITs. Repeat assessment with NITs at 1–2 year intervals should be guided by fibrosis stage and response to treatment [4,203].

Assessments recommended for individuals with diagnosed MASLD by cardiology clinicians [203].

- Use widely available validated serum (eg, FIB-4) and imaging-based noninvasive tests with high negative predictive value to risk-stratify for hepatic fibrosis in individuals with MASLD.
- FIB-4 ≥ 2.67 indicates a high probability of advanced fibrosis, which warrants secondary assessment and/or hepatology referral.
- Combining ≥2 noninvasive tests, using either serum or imaging-based tests, may be considered in patients at intermediate or high risk of hepatic fibrosis.
- Patients stratified as high risk for advanced fibrosis or cirrhosis should be referred to a hepatologist.

Recommendation for systematic cardiovascular screening in all MASLD patients linked to risk-based clinical scenarios [203].

- It is recommended to screen for CVD in all individuals with MASLD, regardless of the presence of traditional atherosclerotic risk factors, with detailed risk-factor evaluation at a minimum.
- Cardiovascular risk assessment should be performed using standard atherosclerotic cardiovascular disease prediction tools.
- Patients with MASLD should be screened annually for type 2 diabetes, hypertension, hyperlipidemia, and overweight/obesity.
- MASLD should be considered a risk-enhancing factor for atherosclerotic CVD.

In MASLD screening, assessment should extend beyond abdominal obesity and body mass index (BMI) to include serum-based indices and imaging modalities for fibrosis staging and surveillance. Importantly, screening for insulin resistance—the principal pathophysiological driver of MASLD—should be performed even in the absence of overweight or obesity [203,204,213,214].

## 5. Integrated Pharmacological Management of the MASLD–CVD Continuum

Substantial challenges complicate the study and therapeutic targeting of the liver–heart axis, largely due to the persistent focus on organ-specific endpoints in clinical trials. MASH studies predominantly emphasize histopathological outcomes, whereas cardiovascular trials rarely incorporate liver-related measures, limiting integrated risk assessment. In addition, concerns regarding hepatic drug metabolism and potential hepatotoxicity often result in the exclusion of patients with liver disease from cardiovascular trials, thereby restricting generalizability. These limitations continue to impede the development of comprehensive, cotargeted therapeutic strategies for MASLD, MASH, and CVD. Within this context, pharmacotherapy must account for potential drug interactions while addressing the shared metabolic substrate underlying both hepatic and cardiovascular disease. Given that MASLD and CVD are driven by common pathophysiological mechanisms, effective treatment strategies should prioritize modulation of key metabolic pathways. Interventions that promote negative energy balance and weight loss are central, as they directly improve adipose tissue dysfunction and lipid metabolism. By targeting these upstream processes, pharmacological approaches can favorably influence insulin resistance, atherogenic dyslipidemia, oxidative stress, and chronic low-grade inflammation. Consequently, an integrated therapeutic framework focused on metabolic pathways is essential for effective cardiac–hepatic cotreatment [12,212,215].

### 5.1. Metabolic Dysfunction-Related Pharmacotherapies

Incretin-based therapies have emerged as a promising treatment modality across MASLD, MASH, and cardiometabolic disease. These include glucagon-like peptide-1 (GLP-1) receptor agonists as well as newer multi-receptor co-agonists, such as dual GLP-1/GIP receptor agonists, GLP-1/glucagon receptor agonists, and triple agonists. By targeting key metabolic pathways, these agents improve insulin sensitivity, diminish hepatic fat content, and promote weight loss through enhanced glucose utilization and suppression of hepatic de novo lipogenesis. Consequently, they attenuate hepatic inflammation and may slow fibrosis progression, while also conferring cardiovascular benefits. Despite their therapeutic potential, widespread implementation is challenged by high costs and a notable side-effect profile, predominantly gastrointestinal in nature [212,216] (Table 3). According to EASL–EASD–EASO Clinical Practice Guidelines on the management of metabolic dysfunction-associated steatotic liver disease (MASLD), in the absence of a formal demonstration of histological improvement in large, well-conducted, phase III trials, glucagon-like peptide 1 receptor agonists (GLP1RA) cannot currently be recommended as MASH-targeted therapies (level of evidence (LoE) 5, strong recommendation, strong consensus). GLP1RAs are safe to use in MASH (including compensated cirrhosis) and should be used for their respective indications, namely type 2 diabetes and obesity, as their use improves cardiometabolic outcomes (LoE 2, strong recommendation, strong consensus). Where available, pioglitazone is safe to use in adults with non-cirrhotic MASH, but given the lack of robust demonstration of histological efficacy on steatohepatitis and liver fibrosis in large phase III trials, pioglitazone cannot be recommended as a MASH-targeted therapy (LoE 2, weak recommendation, consensus) [4].

Fibroblast growth factor 21 (FGF-21) is a hepatokine predominantly produced by the liver that plays a central role in regulating energy homeostasis. FGF-21 analogs enhance lipid metabolism, promote glucose uptake, reduce hepatic fat accumulation, and improve insulin sensitivity, while also demonstrating antifibrotic effects in the liver. Beyond hepatic benefits, these agents exert favorable cardiovascular effects by improving lipid profiles, enhancing vascular function, and attenuating chronic low-grade inflammation [212,217].

### 5.2. Liver-Related Pharmacotherapies

Thyroid hormone receptor-β (THR-β) agonists are liver-directed agents that modulate key metabolic pathways, including hepatic lipid and cholesterol metabolism, gluconeogenesis, and fatty acid oxidation. They represent an emerging therapeutic option for MASLD and MASH, with demonstrated efficacy in reducing hepatic steatosis and improving lipid profiles. Notably, these agents lower circulating triglyceride and LDL cholesterol concentrations; however, direct evidence linking these improvements to reductions in cardiovascular-related events remains limited. From a clinical perspective, potential drug–drug interactions must be carefully considered. THR-β agonists like resmetirom are metabolized via pathways involving CYP2C8, and co-administration with CYP2C8 inhibitors, including the acyl glucuronide metabolite of clopidogrel, may alter drug exposure, necessitating dose adjustments. This is particularly relevant in patients with multimorbidity, especially those with known CVD [212,218,219] (Table 3). According to EASL–EASD–EASO Clinical Practice Guidelines on the management of metabolic dysfunction-associated steatotic liver disease (MASLD), Resmetiron, if approved locally, may be considered for individuals with MASLD who are non-cirrhotic and with documentation of either (A) advanced fibrosis; (B) at-risk steatohepatitis with significant fibrosis (by liver biopsy, when available, or by non-invasive panels validated for that purpose); or (C) risk of adverse liver-related outcomes (e.g., by elastography- or biomarker-defined thresholds) (LoE 3, open recommendation, consensus) [4].

Peroxisome proliferator-activated receptors (PPARs) are nuclear receptors that regulate lipid metabolism, glucose homeostasis, and inflammation. The major isoforms exert tissue-specific effects. PPAR-α (predominantly hepatic) promotes fatty acid β-oxidation, PPAR-β/δ (expressed in skeletal muscle and adipose tissue) modulates energy expenditure, and PPAR-γ (primarily in adipose tissue and immune cells) enhances insulin sensitivity while attenuating inflammation and fibrotic responses. Pioglitazone, a PPAR-γ agonist, is recommended in patients with type 2 diabetes and MASH, where it improves insulin sensitivity and favorably modifies lipid profiles by reducing triglycerides and increasing HDL cholesterol. These effects translate into a reduction in atherosclerotic cardiovascular risk; however, its use is limited by side effects, including weight gain and fluid retention, which may increase the risk of heart failure. Accordingly, pioglitazone should be used with caution and generally avoided in patients with established or high-risk heart failure. To mitigate these limitations, dual and pan-PPAR agonists have emerged as promising alternatives, targeting multiple metabolic pathways simultaneously. These agents can improve hepatic steatosis and fibrosis while also exerting beneficial cardiometabolic effects. However, due to the potential for cardiovascular adverse effects, the use of PPAR-targeted therapies requires careful risk stratification and sustained clinical surveillance [212,220,221,222,223,224] (Table 3).

Fatty acid synthase (FASN) inhibitors target de novo lipogenesis by blocking the conversion of acetylcoenzyme A and malonyl-coenzyme A into long-chain fatty acids. Through suppression of hepatic lipid synthesis, these agents reduce liver steatosis and lower plasma LDL cholesterol levels, while shifting the fatty acid profile toward a higher polyunsaturated-to-saturated fatty acid ratio. These metabolic changes may confer dual benefits for hepatic and cardiovascular health [212,225].

Farnesoid X receptor (FXR) agonists primarily target the liver and intestine, where they regulate bile acid homeostasis and lipid and glucose metabolism. FXR activation reduces hepatic lipid accumulation, suppresses inflammation, and attenuates fibrosis, thereby representing a promising therapeutic choice for MASLD and MASH [212,226] (Table 3).

Acetyl-CoA Carboxylase (ACC) is a key rate-limiting enzyme in hepatic de novo lipogenesis that contributes to lipid accumulation. Pharmacological inhibition of ACC reduces hepatic steatosis and improves biomarkers associated with MASLD and MASH. However, this class of agents may paradoxically increase circulating triglyceride levels. To prevent this metabolic side effect, ACC inhibitors are often combined with diacylglycerol acyltransferase 2 (DGAT2) inhibitors, enabling effective hepatic fat reduction while preserving a more favorable systemic lipid profile [212,227,228].

### 5.3. Cardiovascular-Related Pharmacotherapies

Statins remain the cornerstone of therapy for dyslipidemia, effectively lowering plasma LDL concentration and reducing the risk of major adverse cardiovascular events. The 2022 AHA/ACC/HFSA and 2023 ESC heart failure guidelines recommend high-intensity statins for ischemic heart failure due to underlying coronary disease, but not routinely for non-ischemic phenotypes [229,230]. In chronic coronary disease, the 2023 AHA/ACC CCD and 2024 ESC CCS guidelines issue a Class I recommendation for maximally tolerated high-intensity statins to achieve ≥50% LDL-C reduction, targeting LDL-C levels below 70 mg/dL and 55 mg/dL, respectively [231,232]. In acute coronary syndromes, the 2023 ESC ACS and 2025 ACC/AHA ACS guidelines recommend immediate high-intensity statin therapy (Class I), with early addition of non-statin agents if lipid goals are unmet [233,234]. Beyond their lipid-lowering properties, statins exert pleiotropic effects, including anti-inflammatory, anti-oxidative, and plaque-stabilizing actions. Evidence from large international, multicenter cohort studies indicates that statin therapy is associated with reduced all-cause mortality, fewer liver-related events, a decreased risk of hepatocellular carcinoma, and a slower progression of liver stiffness. These findings support the safe and beneficial use of statins in patients with MASLD who have dyslipidemia or an elevated cardiovascular risk profile [212,235,236,237].

Originally developed for the treatment of type 2 diabetes, sodium–glucose cotransporter-2 (SGLT2) inhibitors have evolved into foundational cardiovascular therapies, demonstrating substantial benefits across the spectrum of heart failure and ischemic heart disease. SGLT2 inhibitors carry a Class I recommendation in the 2022 AHA/ACC/HFSA and 2023 ESC heart failure guidelines for heart failure with reduced ejection fraction (HFrEF), with recommendations extended to Class I/2a in heart failure with preserved ejection fraction (HFpEF) to reduce mortality and heart failure hospitalizations [229,230]. In chronic coronary disease, the 2023 AHA/ACC CCD and 2024 ESC CCS guidelines assign a Class I recommendation for patients with concomitant type 2 diabetes or chronic kidney disease to lower the risk of MACE [231,232]. In acute coronary syndromes, the 2025 ACC/AHA ACS guideline provides a Class I recommendation for initiation before discharge in patients with clinical heart failure or severe left ventricular dysfunction, while recommendations are downgraded to Class 2a/2b in unselected acute patients to attenuate adverse ventricular remodeling [234]. Recently, SGLT2 inhibitors have emerged as promising hepatoprotective agents, demonstrated to reduce hepatic adiposity, improve hepatic insulin resistance, and facilitate meaningful weight loss (Table 3). By promoting glucosuria and reducing systemic glucose levels, these agents mitigate the metabolic drivers of both liver and heart disease. However, clinical implementation requires a cautious, individualized approach. In patients with advanced liver condition or significant renal impairment, the risk/benefit ratio must be carefully weighed due to potential adverse effects, most notably euglycaemic diabetic ketoacidosis and dehydration resulting from osmotic diuresis [212,238,239]. The EASL–EASD–EASO Clinical Practice Guidelines state that while SGLT2 inhibitors lack sufficient evidence to be recommended as direct MASH-targeted therapies, they remain safe to use in MASLD. Consequently, they should be prescribed for their primary metabolic and cardiovascular indications, including type 2 diabetes, heart failure, and chronic kidney disease (LoE 3, strong recommendation, strong consensus) [4].

In the acute setting, the 2023 ESC and 2025 ACC/AHA ACS guidelines mandate an immediate aspirin loading dose followed by low-dose maintenance, typically within a 12-month antiplatelet framework [230,234]. For chronic conditions, the 2023 AHA/ACC CCD and 2024 ESC CCS guidelines require lifelong low-dose aspirin monotherapy (Class I) for secondary prevention in established coronary disease [231,232]. In heart failure, the 2022 AHA/ACC/HFSA and 2023 ESC HF guidelines dictate aspirin use strictly based on an underlying ischemic etiology, omitting it in non-ischemic phenotypes to minimize bleeding risk [229,230]. Emerging evidence suggests it also exerts a beneficial impact on MASLD progression through multiple metabolic and immunomodulatory pathways. Beyond its well-established antiplatelet effects, aspirin mitigates hepatic injury by attenuating chronic low-grade inflammation, inhibiting de novo lipid biosynthesis, and enhancing fatty acid β-oxidation. By targeting these shared inflammatory and metabolic drivers, aspirin represents a dual-benefit strategy in the clinical management of the MASLD-CVD continuum [212,240].

Renin–angiotensin system (RAS) inhibitors, including angiotensin-converting enzyme inhibitors (ACEIs), angiotensin receptor blockers (ARBs), and angiotensin receptor–neprilysin inhibitors (ARNIs), are cornerstone therapies that improve cardiovascular outcomes by enhancing vascular function and reducing systemic afterload. In heart failure (HFrEF), the 2022 AHA/ACC/HFSA and 2023 ESC guidelines mandate RAS inhibitors as a Class I pillar, prioritizing ARNI over ACEI/ARB for mortality reduction [229,230]. For acute and chronic coronary disease, the 2023/2025 ACS and 2023/2024 CCD/CCS guidelines issue a Class I mandate for ACEI (or ARB) therapy if the patient has concurrent LVEF less than 40%, clinical heart failure, hypertension, diabetes, or CKD. In the absence of these specific comorbidities or ventricular dysfunction, routine RAS inhibition remains a Class 2a secondary prevention consideration across all ischemic syndromes [231,232,233,234]. Beyond these effects, these agents exert significant pleiotropic benefits in the context of MASLD; they have been shown to dampen systemic inflammation, improve insulin sensitivity, and potentially attenuate hepatic fibrosis. By disrupting the pro-fibrotic signaling mediated by Angiotensin II, RAS inhibitors represent a critical therapeutic link in mitigating both myocardial remodeling and progressive liver scarring [212,241].

In heart failure (HFrEF), the 2022 AHA/ACC/HFSA and 2023 ESC guidelines mandate MRAs (mineralocorticoid receptor antagonists) as a Class I foundational pillar to reduce mortality and hospitalizations [229,230]. For acute and chronic coronary syndromes, the 2023/2025 ACS and 2023/2024 CCD/CCS guidelines issue a Class I directive for MRA initiation strictly in post-MI patients with an LVEF less than 40% combined with symptomatic heart failure or diabetes [231,232,233,234]. Emerging data suggest these agents may also exert significant hepatoprotective effects by enhancing insulin sensitivity, reducing chronic systemic inflammation, and attenuating liver fibrosis. Clinical evidence indicates that low-dose spironolactone, particularly when combined with vitamin E, significantly decreases the MASLD liver fat score and improves insulin resistance. However, clinical implementation in patients with advanced liver disease requires meticulous monitoring. The metabolic milieu of advanced MASLD often increases the risk of hyperkalemia and renal impairment, potentially complicating the long-term therapeutic regimen. Consequently, a personalized, dose-titrated approach is essential to maximize the cardioprotective benefits of MRAs while minimizing the risk of electrolyte disturbances [212,242,243].

### 5.4. Combination of Liver-Related and Cardiovascular-Related Pharmacotherapies

A strategic combination of pharmacotherapies offers a rational approach to improve hepatic and cardiovascular outcomes simultaneously. Monotherapies often provide organ-specific benefits; liver-directed agents primarily attenuate steatosis, inflammation, and fibrosis, but might have limited impact on cardiovascular risk, whereas cardiovascular-related treatments frequently yield minimal improvements in hepatic pathology. Given the shared metabolic underpinnings of MASLD and CVD, combination regimens that target common pathways, particularly insulin resistance, lipid dysregulation, and chronic inflammation, represent a more effective, integrative strategy [212]. Table 4 shows the phase 3 clinical trials in the MASLD–CVD continuum with hepatic and cardiovascular outcomes.

Metabolic-centered combination approaches may enhance therapeutic efficacy through complementary mechanisms of action, including the following:

➢ GLP-1 receptor agonists + FGF21 analogs: Phase 1b/2a trial of HEC 88473, a GLP-1 and FGF21 dual co-agonist, showed 50% reduction in hepatic fat content over 5 weeks in patients with MASLD + T2DM [244,245].
➢ GLP-1 receptor agonists + THR-β agonists: Used together, these treatments should work synergistically, by targeting both intra- and extra-hepatic factors, although clinical trial evidence is not yet available.
➢ GLP-1 receptor agonists + FXR agonists + ACC inhibitors: Phase 2 trial showed a greater reduction in hepatic fat content, as proven by MRI proton density fat fraction. Despite the similar weight loss being noticed across all drug therapy groups, the combination therapy significantly improved liver biochemistry and non-invasive fibrosis markers [246].
➢ GLP-1 receptor agonists + SGLT2 inhibitors: AMPLITUDE-O trial showed that efpeglenatide significantly reduced the risk of adverse CVD outcomes compared to placebo, regardless of concurrent SGLT2 inhibitor use. The cardiovascular and renal benefits of these agents are well established, but there is a need for further clinical studies to evaluate synergistic effects on liver histological features and long-term liver-related events [247].
➢ PPAR agonists + SGLT2 inhibitors: In the Phase 2 LEGEND trial, after 24 weeks of treatment, HbA1c was significantly reduced by lanifibranor alone, and in combination therapy, significantly improved liver fat, inflammation, fibrosis biomarkers, and maintained stable bodyweight. There was a reduction in the visceral-to-subcutaneous fat ratio [248].

Such combination strategies, by addressing the interconnected pathophysiology of the liver–heart axis, hold promise for more comprehensive disease modification. However, their clinical implementation requires careful evaluation of safety, tolerability, and potential drug–drug interactions.

**Table 4:** Phase 3 clinical trials in the MASLD–CVD continuum: hepatic and cardiovascular outcomes.

Clinical Trial	Clinical Diagnosis	Hepatic Outcomes	Cardiovascular Outcomes	Reference
PIVENS (Pioglitazone, PPAR-γ agonist)	MASH without diabetes	MASH improvement + fibrosis improvement	Direct cardiovascular outcomes not available	[249]
ESSENCE (Semaglutide, GLP-1 receptor agonist)	MASH + F2-F3 fibrosis	MASH improvement without fibrosis worsening Fibrosis improvement without MASH worsening	Direct cardiovascular outcomes not available	[250]
SUSTAIN 6 (Semaglutide, GLP-1 receptor agonist)	Type 2 diabetes + high CV risk	Direct hepatic outcomes not available	↓ risk of MACE (CV death, non-fatal myocardial infarction, or non-fatal stroke) ↓ risk of non-fatal stroke ↓ risk of non-fatal myocardial infarction	[251]
SELECT (Semaglutide, GLP-1 receptor agonist)	Overweight or obesity (no diabetes)	Direct hepatic outcomes not available	↓ risk of MACE (CV death, non-fatal myocardial infarction, or non-fatal stroke)	[252]
STEP-HFpEF (Semaglutide, GLP-1 receptor agonist)	Obesity-related HFpEF	Direct hepatic outcomes not available	Improved heart failure-related symptoms and physical limitations (KCCQ-CSS improvement) Weight loss	[253]
STEP-HFpEF DM (Semaglutide, GLP-1 receptor agonist)	Obesity-related HFpEF + type 2 diabetes	Direct hepatic outcomes not available	Improved heart failure-related symptoms and physical limitations (KCCQ-CSS improvement) Weight loss	[254]
FLOW (Semaglutide, GLP-1 receptor agonist)	Type 2 diabetes + CKD	Direct hepatic outcomes not available	↓ risk of MACE (CV death or heart failure events) ↓ risk of CKD progression	[255]
SOUL (Semaglutide [per os], GLP-1 receptor agonist)	Type 2 diabetes + CVD or CKD	Direct hepatic outcomes not available	↓ risk of MACE (CV death, non-fatal myocardial infarction, or non-fatal stroke)	[256]
LEADER (Liraglutide, GLP-1 receptor agonist)	Type 2 diabetes + high CV risk	Direct hepatic outcomes not available	↓ risk of MACE (CV death, non-fatal myocardial infarction, or non-fatal stroke) ↓ risk of CV death	[257]
NCT02637973 (Empagliflozin, SGLT2 inhibitor)	Well-controlled type 2 diabetes	Liver fat content reduction	Direct cardiovascular outcomes not available	[258]
EMPA-REG OUTCOME (Empagliflozin, SGLT2 inhibitor)	Type 2 diabetes + high CV risk	Direct hepatic outcomes not available	↓ risk of MACE (CV death, non-fatal myocardial infarction, or non-fatal stroke) ↓ risk of CV death ↓ risk of hospitalization for heart failure	[259]
CANVAS (Canagliflozin, SGLT2 inhibitor)	Type 2 diabetes + high CV risk	Direct hepatic outcomes not available	↓ risk of MACE (CV death, non-fatal myocardial infarction, or non-fatal stroke) ↓ risk of hospitalization for heart failure	[260]
DECLARE-TIMI 58 (Dapagliflozin, SGLT2 inhibitor)	Type 2 diabetes + high CV risk or multiple risk factors	Direct hepatic outcomes not available	↓ risk of MACE (CV death, non-fatal myocardial infarction, or non-fatal stroke) ↓ risk of hospitalization for heart failure	[261]
DAPA-HF (Dapagliflozin, SGLT2 inhibitor)	HFrEF (with or without type 2 diabetes)	Direct hepatic outcomes not available	↓ risk of worsening heart failure events ↓ risk of CV death	[262]
EMPEROR-Reduced (Empagliflozin, SGLT2 inhibitor)	HFrEF (with or without type 2 diabetes)	Direct hepatic outcomes not available	↓ risk of CV death or hospitalization for heart failure	[263]
EMPEROR-Preserved (Empagliflozin, SGLT2 inhibitor)	HFpEF (with or without type 2 diabetes)	Direct hepatic outcomes not available	↓ risk of CV death or hospitalization for heart failure	[264]
DELIVER (Dapagliflozin, SGLT2 inhibitor)	HFpEF (with or without type 2 diabetes)	Direct hepatic outcomes not available	↓ risk of CV death or hospitalization for heart failure	[265]
REGENERATE (Obeticholic acid, FXR agonist)	MASH + F2-F3 fibrosis	Fibrosis improvement (≥1 stage) without worsening of MASH	Direct cardiovascular outcomes not available	[266,267]
MAESTRO-NASH (Resmetiron, THR-β agonist)	MASH + F1-F3 fibrosis	MASH resolution without fibrosis worsening Fibrosis improvement without MASH worsening	Direct cardiovascular outcomes not available	[268]
SUMMIT (Tirzepatide, GIP/GLP-1 receptor agonist)	Obesity-related HFpEF	Direct hepatic outcomes not available	↓ risk of CV death or worsening heart failure events	[269]
SURPASS-CVOT (Tirzepatide, GIP/GLP-1 receptor agonist)	Type 2 diabetes + atherosclerotic CVD	Direct hepatic outcomes not available	Noninferior to dulaglutide for MACE (CV death, non-fatal myocardial infarction, or non-fatal stroke)	[270]

Abbreviations: MACE: major adverse cardiovascular events, MASLD: metabolic dysfunction-associated steatosis liver disease, MASH: metabolic dysfunction-associated steatohepatitis, CKD: chronic kidney disease, KCCQ-CSS: Kansas City Cardiomyopathy Questionnaire-Clinical Summary Score, HFpEF: heart failure with preserved ejection fraction, HFrEF: heart failure with reduced ejection fraction, ↓: decrease.

## 6. Conclusions and Perspectives

MASLD serves as the hepatic manifestation of a systemic metabolic disorder, initiated by the accumulation of excess lipids driven by high dietary fatty acid intake and accelerated de novo lipogenesis. This triglyceride buildup precipitates lipotoxicity, which triggers a cascade of hepatocyte injury, immune cell activation, and mitochondrial dysfunction. Within the mitochondria, the excessive generation of ROS serves as a pivotal second hit, activating the production of inflammatory cytokines that drive disease progression.

Beyond lipid dysregulation, impaired hepatic insulin signaling drives systemic hyperglycemia accompanied by compensatory hyperinsulinemia. This state promotes selective activation of lipogenic pathways, further stimulating de novo lipogenesis and reinforcing a self-perpetuating cycle of metabolic dysfunction. At the cellular level, persistent ROS generation overwhelms the body’s antioxidant defenses and depletes hepatic GSH stores, thereby exacerbating cellular redox imbalance. These intracellular disturbances propagate systemically, contributing to atherogenic dyslipidemia and the release of pro-inflammatory mediators. Concurrently, gut dysbiosis and increased intestinal permeability amplify systemic inflammation through the translocation of microbial products, further integrating metabolic and immune dysfunction. As hepatic GSH depletion translates into reduced circulating antioxidant capacity, plasma GSH levels decline, weakening systemic redox buffering. The resulting oxidative stress represents the key connection between MASLD progression and CVD development. Beyond direct cellular damage, ROS alter protein functions via post-translational thiol oxidation, disrupting critical signaling pathways involved in vascular and cardiac homeostasis, inflammation, and metabolism. Collectively, these processes promote endothelial dysfunction, atherogenesis, and cardiovascular remodeling. In this context, strategies aimed at restoring redox balance, potentially including targeted antioxidant approaches, may complement standard therapeutic interventions and mitigate disease progression.

There is a growing body of evidence supporting the utility of NITs for the detection and risk stratification of MASLD. The long-term objective is to integrate a non-invasive approach into routine clinical practice to facilitate early identification of individuals at increased cardiometabolic risk who may benefit from targeted cardiological evaluation. By capturing the shared pathophysiological substrate between hepatic and cardiovascular disease, such strategies hold potential to improve risk stratification and enable timely prevention of adverse cardiovascular outcomes.

## Figures and Tables

**Table 1:** Fatty acid oxidation, ER stress, and mitochondrial dysfunction in MASLD.

Fatty Acid Oxidation-Related Experiment	Key Findings
in vivo: siRNA-mediated depletion of Methylation-Controlled J (MCJ) protein—transmembrane inner mitochondrial membrane protein	reduces liver steatosis and fibrosis → increased β-oxidation [49,50,51]
in vitro: Farnesol (key element in cholesterin biosynthetic pathway) is dephosphorylated and transforms into non-sterol isoprenoid farnesol (FOH)	modulates fatty acid oxidation reduces expression of fatty acid synthesis genes via farnesoid X receptor (FXR) and PPAR [52]
in vivo: caspase 1 and neutrophil elastase inhibition	beneficial roles on fatty acid oxidation [53]
in vivo: PPARα activity modulation via: ➢ PI3K ➢ PARP1 (poly-ADP-ribose polymerase 1) ➢ Zinc finger protein 300	fatty acid oxidation reduction [54,55,56]
in vivo: SMAD signaling	regulates the expression of β genes, which are involved in the induction of lipid accumulation [57]
**ER stress-related experiment**	**Key findings**
in vivo: FOXA3 protein coding gene (Forkhead box A3 = member of FOXA family): ➢ XBP1c (transcriptional factor) ➢ high carbonhydrate diet	interferes with the modulation of FOXA3 activity, increases Ca^2+^ influx, and upregulates ER stress markers [47]. This process can be reversed by 4-PBA (4-phenylbutyric acid) → reduced mRNA expression of lipogenic genes and increased lipolytic gene expression [58]
**Mitochondrial dysfunction-related experiment**	**Key findings**
in vivo: Ant 2 protein (inner mitochondria membrane protein)	experimentally inhibited → improvement in hepatic steatosis and insulin resistance, but no influence on inflammation [59]
in vivo: Perilipin 5 (lipid droplet coat protein)	high expression in brown adipose tissue improves insulin sensitivity and overall mitochondrial function [60]
human: Sam 50 protein (outer mitochondria membrane protein) modulated by sorting and assembly machinery (SAM) complex protein (SAMM50)	Sam 50 protein has a role in: ➢ regulation of oxidative activity ➢ mitochondrial morphology ➢ mitophagy decreased level of SAMM50 in encoding Sam50→ decreased rate of fatty acid oxidation [61]
in vitro: mitochondrial membrane potential role in hyperglycemic status	hyperglycemia led to: ➢ hyperpolarization of the membrane ➢ mitochondrial dysfunction ➢ high risk for collapse of potential membrane with signals towards apoptosis [62]
in vivo: mixture of nutraceuticals (vit.E, vit.D, olive dry extract, cinnamon dry extract)	increased respiratory chain activity + mitochondrial ATP production [49,63]
in vitro: role of polyphenols	improves mitochondrial function by promoting SIRT1 (sirtuin 1) activity and deacetylation of PPAR-γ coactivator-1α (PGC-1) [49,64]
in vivo: Astaxanthin	improves mitochondrial function [49,65]
in vitro+ in vivo: Chemokine receptor CXCR3	inhibition of CXCR3 led to: ➢ restoration of mitochondrial function ➢ inhibition of mitochondrial-dependent apoptosis [49,66,67]
in vivo: neutrophil activity	experimental depletion restores the function and number of mitochondria [49,68]

**Table 3:** Pharmacological therapies of the MASLD–CVD continuum.

Medication Group	Mechanisms	Liver Benefits	Fibrosis Outcome	Cardiac Benefits
PPAR agonists (TZDs)	↓ hepatic and peripheral insulin resistance	↓ liver fat content	↓ fibrosis progression	↓ risk of atherosclerosis (↑ risk of heart failure)
GLP-1 receptor agonists	↑ insulin secretion ↓ appetite	weight loss ↓ liver fat content ↑ hepatocyte regeneration	↓ fibrosis	↓ risk of MACE (CV death, non-fatal myocardial infarction, or non-fatal stroke)
SGLT-2 inhibitors	↓ renal glucose absorption ↑ glucosuria	weight loss ↓ blood glucose ↓ liver fat content	↓ fibrosis	↓ risk of CV death ↓ the risk of heart failure improves cardiac function
FXR agonist (OCA)	improved bile acid metabolism	↓ liver fat content	↓ fibrosis progression	no available information
THR-β agonist (Resmetiron)	improved lipid metabolism	↓ liver fat content ↓ inflammation markers	↓ fibrosis	cardiovascular protective effects, by lowering circulating plasma lipid levels (MAESTRO trial): ↓ MACE
Dual GLP-1 and GIP receptor agonist (Survotide/Tirzepatide)	weight loss	↓ liver fat content weight loss	↓ liver fibrosis	↓ CVD risk

Abbreviations: TZDs: thiazolidinediones, CVD: cardiovascular disease, CV: cardiovascular, GLP-1: glucagon-like peptide-1, MACE: major adverse cardiovascular events, THR-β: Selective Thyroid Hormone Receptor-Beta (TRβ) agonist, OCA: obeticholic acid, FXR: farnesoid X receptor, GIP: glucose-dependent insulinotropic polypeptide, MASLD: metabolic dysfunction-associated steatosis liver disease,↓: decrease, ↑: increase.

## Data Availability

No new data were created or analyzed in this study. Data sharing is not applicable to this article.

## Abbreviations

The following abbreviations are used in this manuscript:

MASLD	Metabolic dysfunction-associated steatotic liver disease
MRI	Magnetic resonance imaging
MetS	Metabolic syndrome
NAFLD	Non-alcoholic fatty liver disease
ROS	Reactive oxygen species
sdLDL	Small dense low-density lipoprotein
TNF-α	Tumor necrosis factor-α
IL-6	Interleukin-6
CRP	C-reactive protein
MACE	Major adverse cardiovascular events
VLDL	Very low-density lipoprotein
HDL	High-density lipoprotein
IDL	intermediate-density lipoprotein
DAMPs	Damage-associated molecular patterns
TLRs	Toll-like receptors
ApoC3	Apolipoprotein C3
NOD	Nucleotide-binding oligomerization domain-like receptors
NLRP3	NOD-like receptor family, pyrin domain-containing protein 3
PAMPs	Pathogen-associated molecular patterns
SREBP-1C	Sterol regulatory element-binding protein 1c
ChREBP	Carbohydrate-responsive element-binding protein
PAI-1	Plasminogen activator inhibitor-1
NF-κB	Nuclear factor κB
NADPH	Nicotinamide adenine dinucleotide phosphate
FGF 21	Fibroblast growth factor 21
PPAR-γ	Peroxisome proliferator-activated receptor gamma
AMP	adenosine monophosphate
PCG	Polycomb group
JAK	Janus kinase
STAT	Signal transductor and activator of transcription
PI3K	Phosphoinozitol 3 kinase
Akt	AKT serine/threonine kinase, also called protein kinase B (PKB)
HOMA-IR	Homeostasis model assessment method for insulin resistance
ADMA	Asymmetric dimethyl arginine
VEGF	Vascular endothelial growth factor
MAMPs	Microbe-associated molecular patterns
LPS	Lipopolysaccharide
NASH	Non-alcoholic steatohepatitis
TMAO	Trimethylamine N-oxide
GLP-1	Glucagon-like peptide-1
HNE	4-hydroxy-2-nonenal
8-OHdG	8-hydroxydeoxyguanosine
DNA	Deoxyribonucleic acid
IFN-γ	Interferon-γ
NK	Natural killer
KEAP1	Kelch-like ECH-associated protein 1
Nerf2	nuclear factor erythroid 2-related factor
MCJ	Methylation-controlled J protein
siRNA	Small interference ribonucleic acid
FXR	Farnesoid X receptor
PARP1	poly-(ADP ribose) polymerase 1
ER	endoplasmic reticulum
PERK	Protein kinase R-like endoplasmic reticulum kinase
eIFα	Eukaryotic initiation factor α
ATF4	Activating transcription factor 4
IRE1	Inositol-requiring enzyme 1
XBP 1	X-box binding protein 1
FOXA3	Forkhead box A3
4-PBA	4-phenylbutiryc acid
mRNA	Messenger ribonucleic acid
Ant 2	Adenine nucleotide translocase 2
SAM	Sorting and assembly machinery
ATP	Adenosine triphosphate
SIRT1	Sirtuin 1
FFA	Free fatty acids
TG	Triglyceride
MASH	Metabolic dysfunction-associated steatohepatitis
IRS-1	Insulin receptor substrate-1
eNOS	Endogen nitric oxide synthase
HFpEF	Heart failure with preserved ejection fraction
TGF-β	Transforming growth factor-β
CVD	Cardiovascular disease
SCFAs	Short-chain fatty acids
MAPK	Mitogen-activated protein kinase
PKC	Protein kinase C
ASAT	Aspartate-aminotransferase
ALAT	Alanine-aminotransferase
γ-GT	γ-glutamyl transferase
AASLD	American Association for the Study of Liver Disease
NAS	NAFLD activity score
NIT	Non-invasive test
APRI	Aspartate transaminase to platelet ratio index
ELF	Enhanced Liver Fibrosis
VCTE	Vibration-controlled transient elastography
MRE	Magnetic resonance elastography
